# Comparative genomics of *Leuconostoc lactis* strains isolated from human gastrointestinal system and fermented foods microbiomes

**DOI:** 10.1186/s12863-022-01074-6

**Published:** 2022-08-02

**Authors:** Ismail Gumustop, Fatih Ortakci

**Affiliations:** BioEngineering Department, Faculty of Life and Natural Sciences, Abdullah Gul University, Kayseri, TR Turkey

**Keywords:** *Leuconostoc lactis*, Comparative genomics, Lactic acid bacteria, Prophage, CRISPR-Cas, Bacteriocin

## Abstract

**Background:**

*Leuconostoc lactis* forms a crucial member of the genus *Leuconostoc* and has been widely used in the fermentation industry to convert raw material into acidified and flavored products in dairy and plant-based food systems. Since the ecological niches that strains of *Ln. lactis* being isolated from were truly diverse such as the human gut, dairy, and plant environments, comparative genome analysis studies are needed to better understand the strain differences from a metabolic adaptation point of view across diverse sources of origin. We compared eight *Ln. lactis* strains of 1.2.28, aa_0143, BIOML-A1, CBA3625, LN19, LN24, WIKIM21, and WiKim40 using bioinformatics to elucidate genomic level characteristics of each strain for better utilization of this species in a broad range of applications in food industry.

**Results:**

Phylogenomic analysis of twenty-nine *Ln. lactis* strains resulted in nine clades. Whole-genome sequence analysis was performed on eight *Ln. lactis* strains representing human gastrointestinal tract and fermented foods microbiomes. The findings of the present study are based on comparative genome analysis against the reference *Ln. lactis* CBA3625 genome. Overall, a ~ 41% of all CDS were conserved between all strains. When the coding sequences were assigned to a function, mobile genetic elements, mainly insertion sequences were carried by all eight strains. All strains except LN24 and WiKim40 harbor at least one intact putative prophage region, and two of the strains contained CRISPR-Cas system. All strains encoded Lactococcin 972 bacteriocin biosynthesis gene clusters except for CBA3625.

**Conclusions:**

The findings in the present study put forth new perspectives on genomics of *Ln. lactis* via complete genome sequence based comparative analysis and further determination of genomic characteristics. The outcomes of this work could potentially pave the way for developing elements for future strain engineering applications.

**Supplementary Information:**

The online version contains supplementary material available at 10.1186/s12863-022-01074-6.

## Background

Genus *Leuconostoc* (*Ln*) is comprised of 17 species of *Ln. mesenteroides* (divided into *Ln. mesenteroides*, *Ln. dextranicum*, and *Ln. cremoris*), *Ln. pseudomesenteroides, Ln. citreum, Ln. gelidum, Ln. carnosum, Ln. kimchii, Ln. fallax, Ln. inhae, Ln. palmae, Ln. miyukkimchii, Ln. rapi, Ln. falkenbergense, Ln. holzapfelii, Ln. litchii, Ln. suionicum, Ln. garlicum, Ln. lactis* [1]. As of 2017 the species *suionicum* has been designated, previously considered subspecies of *Ln. mesenteroides* [2].

*Ln. lactis* is a lactic acid bacterium (LAB) that naturally exists in diverse ecological environments and is commonly pertain to food fermentations. The isolation sources of this species are various environments, including cheese, whey, cucumber fermentation brine, kimchi, and the human gut [3, 4].

*Ln. lactis* is a gram ( +), catalase (-), cocci, facultative anaerobic, heterofermentative, non-motile, non-spore forming LAB carrying intrinsic vancomycin resistance [5, 6]. Certain *Ln. lactis* strains are able to produce buttery flavor metabolites for example diacetyl and acetoin at low pH. Thus, they could be utilized in fermented dairy foods [7, 8]. Moreover, some *Leuconostoc* strains could convert carbohydrates such as sucrose to dextran exopolysaccharide [9]. Due to the heterofermentative lifestyle of *Ln. lactis,* it produces equimolar of lactate, ethanol, and carbon dioxide upon fermenting a mole hexose sugar (glucose and galactose), in the absence of an external electron acceptor, through pentose phosphate pathway (PPP) also known as 6-phosphogluconate/phosphoketolase pathway [10]. However, when an external electron acceptor such as acetaldehyde or pyruvate is available in the microenvironment, this organism could convert sugars primarily into lactate, acetate and CO_2_ to maintain redox balance by reoxidizing NADH to NAD^+^ which was reduced to NADH from NAD^+^ in the upper half of PPP. This conversion into acetate produces one additional ATP thus it is more productive for the cell compared to ethanol route also called salvage shunt [5, 11].

The LAB is reported to show high adaptation to specific microbiological niches and carry smaller genomes as opposed to other bacteria because of reducing genome which is the consequence of their effort to maintain only the required number of crucial genes necessary for micro-niche specific survivability. Even though *Ln. lactis* genome is proportionally small; it has to maintain the capability of rapid and continuous evolution with its essential ecosystem via horizontal gene transfer of plasmids or transduction by phage infectivity [12–14]. Moreover, in order to maintain its viability and grow in a changing and highly specific ecosystem, LAB has to balance the maintenance of a strong immune system against bacteriophages, transmissible DNA elements, exogenous plasmids or transposases [14–16].

Several *Leuconostoc* species such as *carnosum* and *mesenteroides* have been evaluated by comparative genomic analysis [2, 17]. Although isolation source of *Ln. lactis* is reportedly diverse, metabolic potentials of *Ln. lactis* strains have not been subjected to extensive genomic research. Therefore, information on the species population dynamics and genomic diversity in various ecological systems such as kimchi, fermented cucumber brine, human gut or dairy is scarce if available at all [4]. To our knowledge, the present work is the first in-depth comparative study of *Ln. lactis* genomics and diversity in the human gastrointestinal tract and fermented foods microbiomes.

## Result

### General genome features

Whole-genome sequence statistics of thirty-three *Ln. lactis* strains extracted from NCBI Genbank [18] are shown in Table 1.

**Table 1:** Whole-genome sequence statistics of thirty-tree *Ln. lactis* strains

Strain	Assembly Accession Number	Isolation Source	Sequencing Technology	Size(Mb)	GC%
CBA3622	GCA_007954625.1	Kimchi	PacBio RSII	1.78764	42.9
WiKim40	GCA_001698145.1	Kimchi	PacBio	1.78807	43.1
CBA3625	GCA_007954605.1	Kimchi	PacBio RSII	1.79161	43.3
CBA3626	GCA_007954665.1	Kimchi	PacBio RSII	1.83981	43.1
UBA8811	GCA_003529125.1	Food	Illumina MiSeq	1.57941	43.5
JCM 6123	GCA_019656035.1	Milk	Illumina NovaSeq	1.61525	43.5
NBRC 12455	GCA_006539105.1	Not available	Illumina HiSeq 1000	1.64349	43.5
JCM 6123	GCA_014651235.1	Not available	Illumina HiSeq X Ten	1.64888	43.3
MSK.22.141	GCA_020708975.1	Fecal sample	Illumina HiSeq	1.68437	43.4
KACC 91922	GCA_000709265.1	Kimchi	Illumina MiSeq	1.68817	43.4
MSK.22.137	GCA_020708945.1	Fecal sample	Illumina HiSeq	1.69008	43.4
1.2.28	GCA_018993775.1	Cucumber fermentation brine	Illumina	1.71216	43.4
KCTC 3773	GCA_000179875.1	Not available	454	1.72068	42.9
LN19	GCA_002092595.1	Dairy	Illumina MiSeq	1.72439	42.9
LN24	GCA_002092695.1	Dairy	Illumina MiSeq	1.72466	42.9
aa_0143	GCA_004167235.1	Stool	Illumina HiSeq	1.73814	43.2
AV1n	GCA_009795665.1	Fruit	Illumina NovaSeq	1.73819	43.2
CCK940	GCA_002287365.1	Kimchi	PacBio	1.74151	43.3
BIOML-A1	GCA_009678855.1	Fecal material [ENVO:00002003]	Illumina NextSeq	1.748	43.1
WIKIM21	GCA_001411775.1	Kimchi	454	1.76143	43.1
SBC001	GCA_014050705.1	Green onion	PacBio RSII	1.83516	43.1
KCTC 3528	GCA_000185085.2	Not available	454 GS Titanium	2.0112	42.6
UBA8466	GCA_003521925.1	Terrestrial	Illumina HiSeq 2500	1.21451	43.2
UBA5657	GCA_002420925.1	Metal	Illumina	1.27472	42.6
UBA5028	GCA_002416225.1	Metal/plastic	Illumina	1.30503	43.1
UBA6751	GCA_002453615.1	Metal/plastic	Illumina	1.5482	43.2
UBA4605	GCA_002386625.1	Metal	Illumina	1.55071	43.7
UBA4610	GCA_002386555.1	cloth	Illumina	1.56779	43.5
UBA5566	GCA_002425565.1	Metal	Illumina	1.57241	43.8
UBA5570	GCA_002425485.1	Wood	Illumina	1.60419	43.5
UBA7653	GCA_002483535.1	Metal	Illumina	1.60507	43.7
1001262B_160229_C9	GCA_015551285.1	Stool	Illumina HiSeq	1.65714	43.6
1001095IJ_161003_G5	GCA_015553465.1	Stool	Illumina HiSeq	1.67719	43.3

Whole-genome statistics of each of thirty-three strains show genome sizes between 1.21 Mb and 2.02 Mb (average 1.66 Mb). GC content is between 42.6% and 43.8% (average 43.3%)

**Fig. 1:**
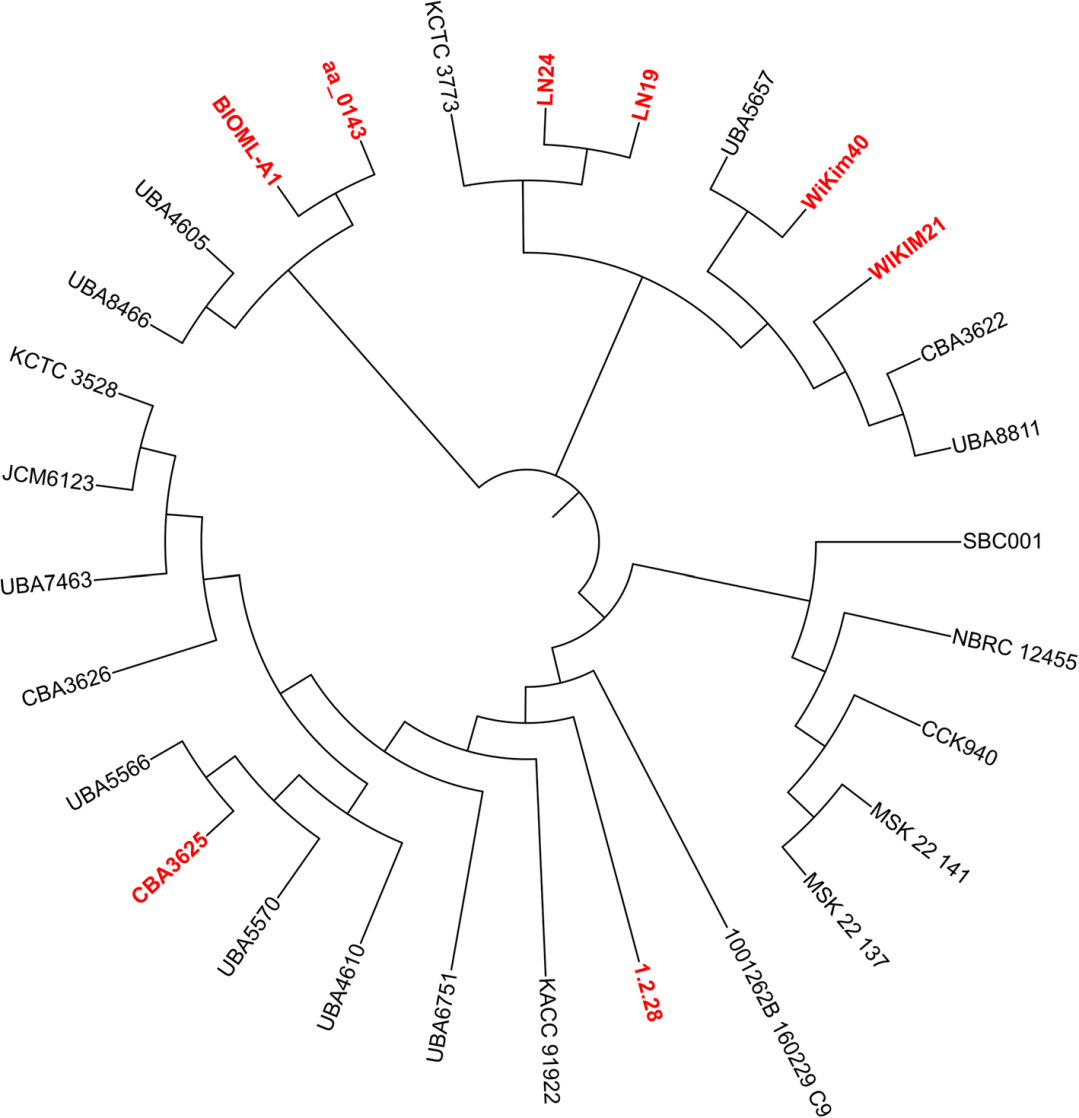
Hierarchical clustering tree of multiple sequence comparison based on phosphoglucomutase gene

The *Leuconstoc lactis* strains studied in the present study were comparatively evaluated using genomic analysis. Twenty-nine strains including the type-strain CBA3625 (Table 1) were chosen for comparative genomic analysis based on phosphoglucomutase gene (Fig. 1). A phylogenetic analysis of 29 strains carrying complete phosphoglucomutase gene was performed based on the nucleotide sequence alignment (Fig. 1). Four strains were eliminated because of truncated or absence of gene of interest. The phylogenetic tree revealed the formation of nine distinct clades. Human gastrointestinal isolates of BIOML-A1 and aa_0143 share the first clade, whereas dairy isolates of LN19, LN24, and kimchi isolates of WIKIM21, WiKim40, and CBA3622 share the second clade. The third clade was composed of green onion and kimchi isolates of SBC001 and CCK940, respectively. NBRC 12455, MSK.22.141, and MSK.22.137 share the same clade however, the isolation sources were not found. Stool isolate of 1001262B_160229_C9, cucumber fermentation brine isolate of 1.2.28, kimchi isolate of KACC 91922, and UBA6751 (isolation source is not available) are located on the fourth clade to seventh clade, respectively. The isolation source of the last two clade members was not available except CBA3625, which was isolated from kimchi (Fig. 1). Interestingly, the kimchi isolate of *Ln. lactis* CBA3625 was part of the eighth clade containing the strains of UBA5570 and UBA5566 isolated from wood and metal, respectively.

### Comparative genomics of *Ln. lactis*

**Fig. 2:**
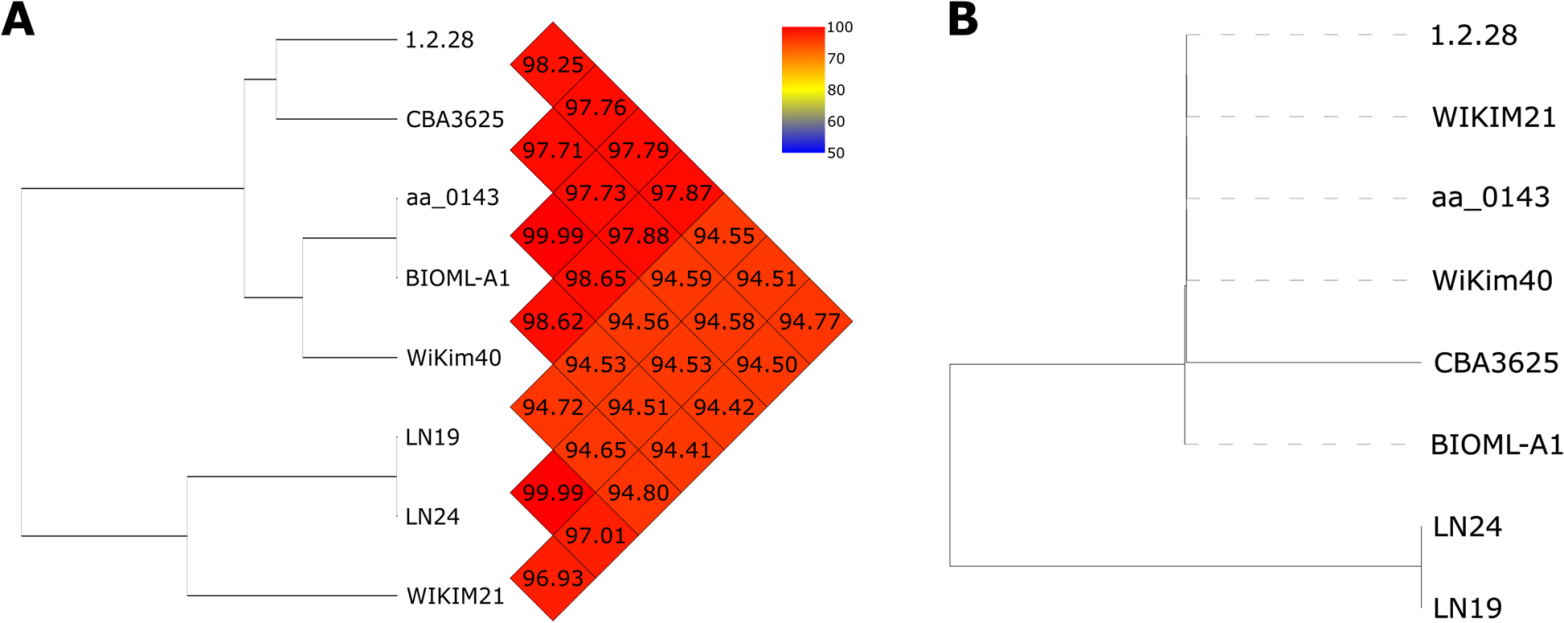
**A** Average OrthoANI nucleotide sequence based phylogenetic tree. **B** 16S rDNA nucleotide sequence based phylogenetic tree of eight *Leuconostoc lactis* strains

**Fig. 3:**
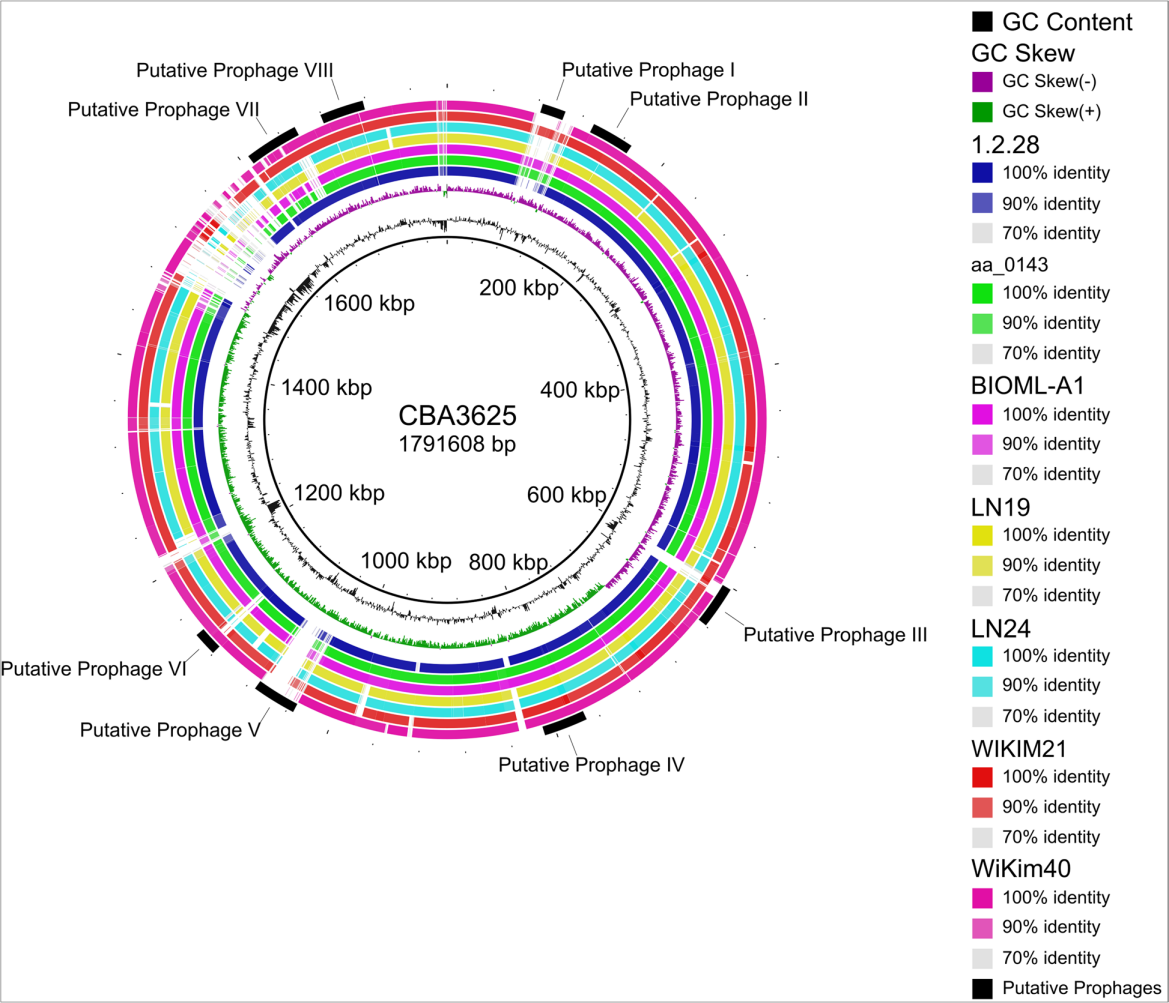
Whole-genome based BLAST comparison of eight *Ln. lactis* strains against reference strain CBA3625

Next, we selected eight strains to conduct whole-genome nucleotide sequence comparisons. The genomes were chosen for further analysis were: BIOML-A1 (fecal sample), aa_0143 (stool), LN24 (dairy), LN19 (dairy), WiKim40 (kimchi), WIKIM21 (kimchi), 1.2.28 (cucumber fermentation brine), and CBA3625 (kimchi). Genomes of these strains were picked as a representative set of phylogenies shown in Fig. 1 and are highlighted in red. These strains were isolated from either fermented foods or the human gastrointestinal tract and range in size from 1.71 Mb to 1.79 Mb. The GC-content of each individual strain ranges between 42.9% and 43.4%. The OrthoANI and 16S rDNA sequence-based phylogenetic trees are shown in Fig. 2. The whole-genome analysis of eight strains was carried out using BRIG (Fig. 3), whole-genome sequence-based phylogenetic tree (Figure S1) and progressive Mauve (Figure S2).

Average OrthoANI nucleotide sequence based phylogenetic tree generated four clades. The member of the clade-one consists of WIKIM21 only. LN24 and LN19 comprised the second clade. WiKim 40, BIOML-A1, and aa_0143 form the third clade. The last clade members were CBA3625 and 1.2.28 (Fig. 2A). The phylogenetic tree of all strains clustered based on 16S rDNA shows two distinct clusters (Fig. 2B). Dairy originated *Ln. lactis* LN19 and LN24 form a separate clade from the remaining strains located on the second cluster from bottom to up (Fig. 2B).

Notably, 1.2.28, WiKim40, and BIOML-A1 share the highest sequence identity against the reference genome CBA3625 (Fig. 2A). Similar results were also seen in the whole genome sequence based phylogenetic tree that the closest neighbors to CBA3625 were 1.2.28, WiKim40, and BIOML-A1 (Figure S1).

Figure 3 shows whole-genome based BLAST comparison of all strains against reference strain CBA3625. BRIG image shows alignment of eight *Ln. lactis* strains and their GC content and GC skews. Four regions lacking significant coverage were identified as putative prophages. The first two pronounced gaps on the genome alignment identified as putative prophage I and III were between 83.8 Kb — 104.5 Kb, and 131.4 Kb — 168.8 Kb, respectively. The largest gap seen at 1.03 Mb — 1.07 Mb was marked as putative prophage V. The last gap positioned between 1.12 Mb — 1.14 Mb was identified as putative prophage VI (Fig. 3, Table 2).

**Table 2:** Prophages predicted in eight *Leuconostoc lactis* strains using PHASTER

Strain	Region	Length	Completeness	Score	# Total Proteins	Start	End	Most Common Phage	GC %
1.2.28	1	37.3 Kb	intact	100	49	131,353	168,750	PHAGE_Strept_PH10_NC_012756(5)	40.96%
BIOML-A1	1	38.4 Kb	intact	150	45	1,679,270	1,717,753	PHAGE_Strept_A25_NC_028697(7)	41.34%
CBA3625	1	20.7 Kb	intact	110	19	83,776	4494	PHAGE_Strept_PH15_NC_010945(6)	41.70%
CBA3625	3	38.6 Kb	intact	150	49	1,034,170	1,072,836	PHAGE_Lactob_Sha1_NC_019489(9)	40.32%
LN19	2	47.3 Kb	intact	150	31	1,607,348	1,654,708	PHAGE_Lactoc_bIL309_NC_002668(6)	39.77%
WIKIM21	1	37.4 Kb	intact	150	54	601,810	639,287	PHAGE_Strept_A25_NC_028697(7)	40.97%
WIKIM21	2	21 Kb	intact	100	22	1,120,379	1,141,394	PHAGE_Strept_A25_NC_028697(4)	41.34%
LN19	3	11.4 Kb	questionable	70	9	1,712,884	1,724,361	PHAGE_Lactob_phiAT3_NC_005893(2)	37.11%
LN24	3	12 Kb	questionable	90	14	1,635,922	1,647,925	PHAGE_Lactoc_bIL309_NC_002668(6)	38.61%
LN24	4	9.6 Kb	questionable	80	15	1,715,002	1,724,631	PHAGE_Paenib_PBL1c_NC_048689(2)	38.37%
WiKim40	2	32.8 Kb	questionable	70	21	1,652,645	1,685,487	PHAGE_Lactob_phiAT3_NC_005893(2)	38.60%

**Fig. 4:**
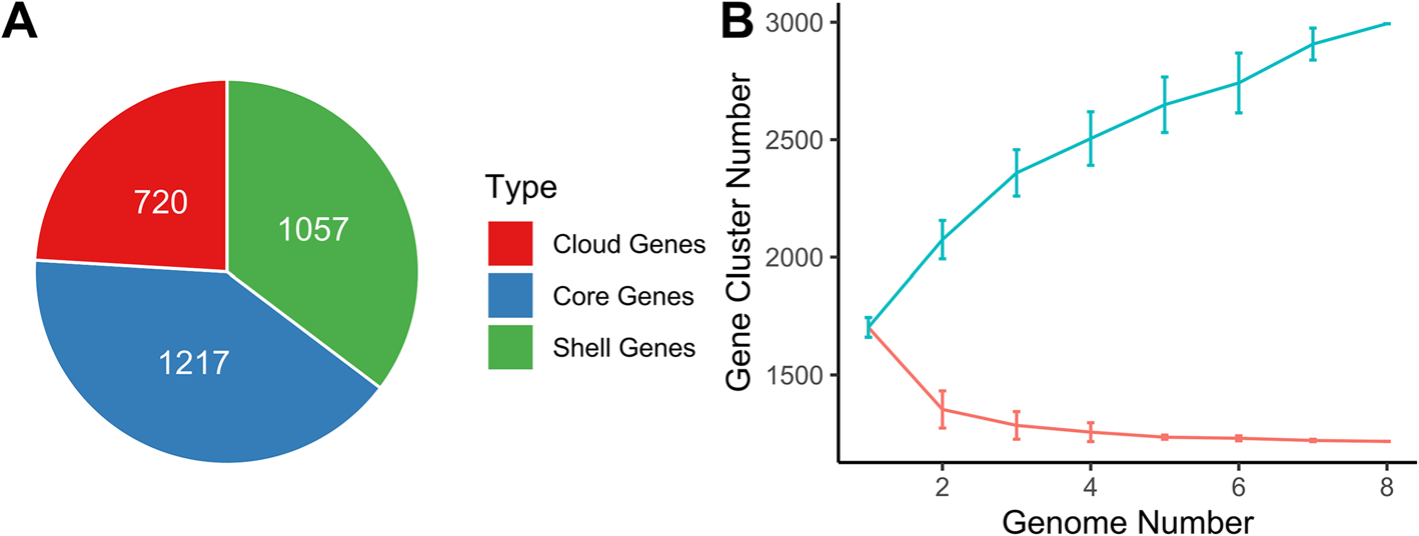
(**A**) Distributions of coding sequences found in *Ln. lactis* pan-genome: Core genes (green), shell genes (blue), cloud genes (red) in chromosome. (**B**) Estimation of the pan-genome (blue) and the core genome (red) of eight *Ln. lactis* strains by including genomes one by one. R programming [20] language and ggplot2 [21] package was used to plot the graphics

For the characterization of genomic conservation between all isolates related to pan- and core genomes, overall coding potential (i.e. pangenome) was determined. It was observed that 40.7% of entire genes are conserved within 95% BLASTP identity (Fig. 4A). Of the 2994 total CDS, 1217 were shared by entire eight strains, which represent the core genome. The accessory genome also called the non-core genome, contained 1777 total CDS, perhaps determining fundamental differences of phenotypic traits across different strains [19].

Interestingly, a considerable number of sequences without function prediction (hypothetical genes) was found across all *Ln. lactis* strains ranging from 34 to 39% (37% on average). These *Ln. lactis* genomes are potential candidates for further functional annotation studies.

**Fig. 5:**
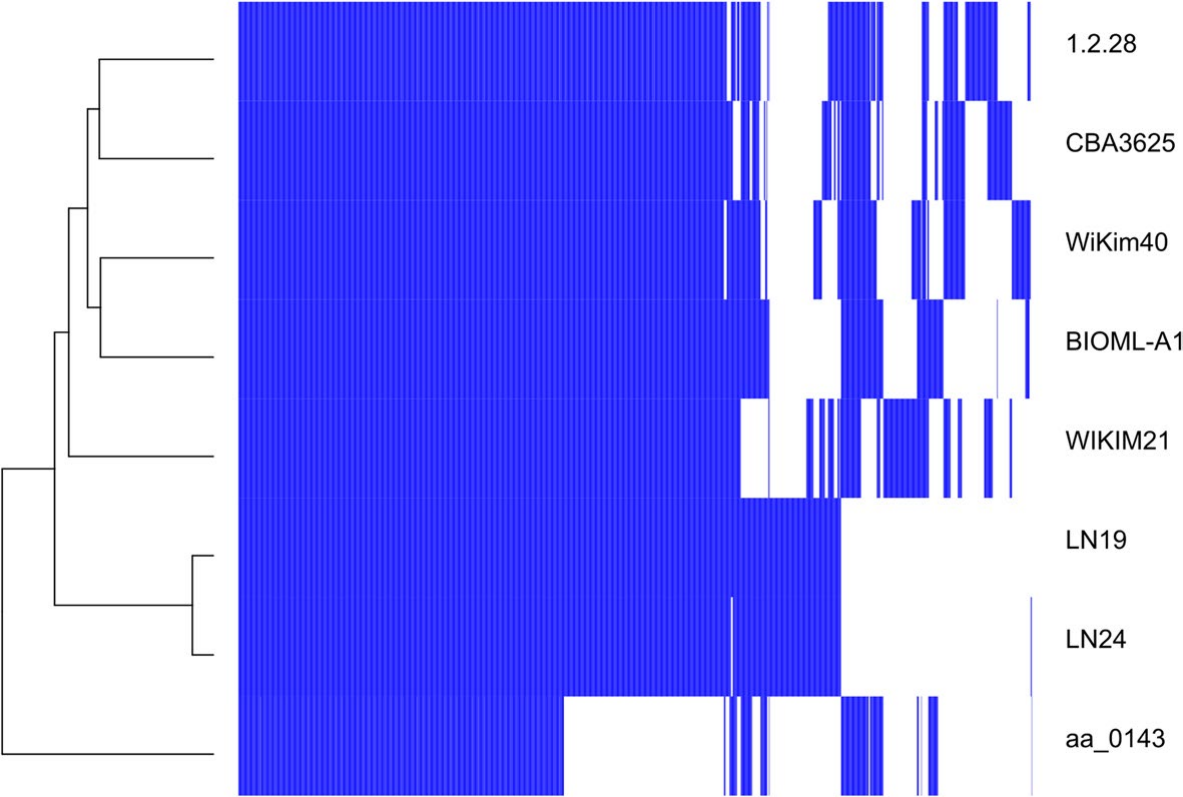
Phylogenetic tree based on gene absence-presence and gene cluster matrix comparing the similarity between putative coding sequences. R programming language was used to create the heatmap [20]

Four distinct clusters emerged after clustering by gene absence/presence matrix (Fig. 5). Cluster 1 only consists of aa_0143 showing the highest percent identity, with cluster 2 composed of LN19 and LN24. Cluster 3 consists of only WIKIM21 and reveals the highest percent identity with BIOML-A1 and WiKim40. There are two sub-clusters within the last cluster which contains 1.2.28 and CBA3625, and WiKim40 and BIOML-A1, respectively.

**Fig. 6:**
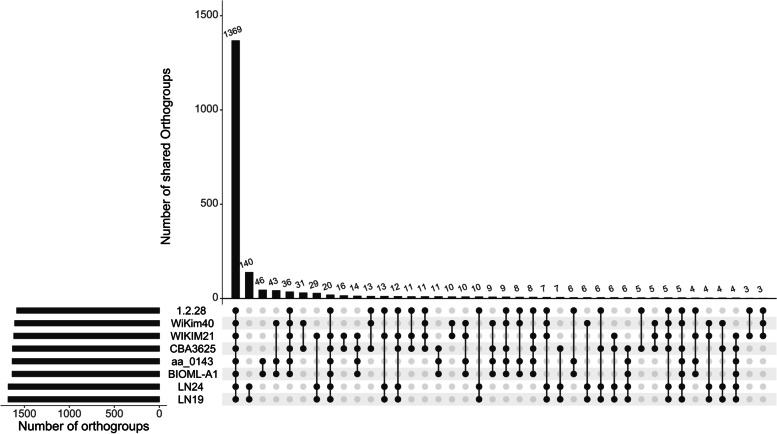
The upSet plot shows the number of orthogroups of each strain and the number of shared orthogroups among the strains with bar charts. UpSetR [22]package in R programming language [20] was used to draw the figure

Figure 6 shows the upSet plot of the number of shared orthogroups of each strain and the number of shared orthogroups among the strains with bar charts. The number of shared orthogroups in all strains was 1369. LN19 and LN24 have the highest number of shared orthogroups among all strains tested. Next, aa_0143 and BIOML-A1 share the 46 orthogroups. aa_0143, BIOML-A1, and WiKim40 share 43 orthogroups.

### Functional characteristics of *Ln. lactis*

**Fig. 7:**
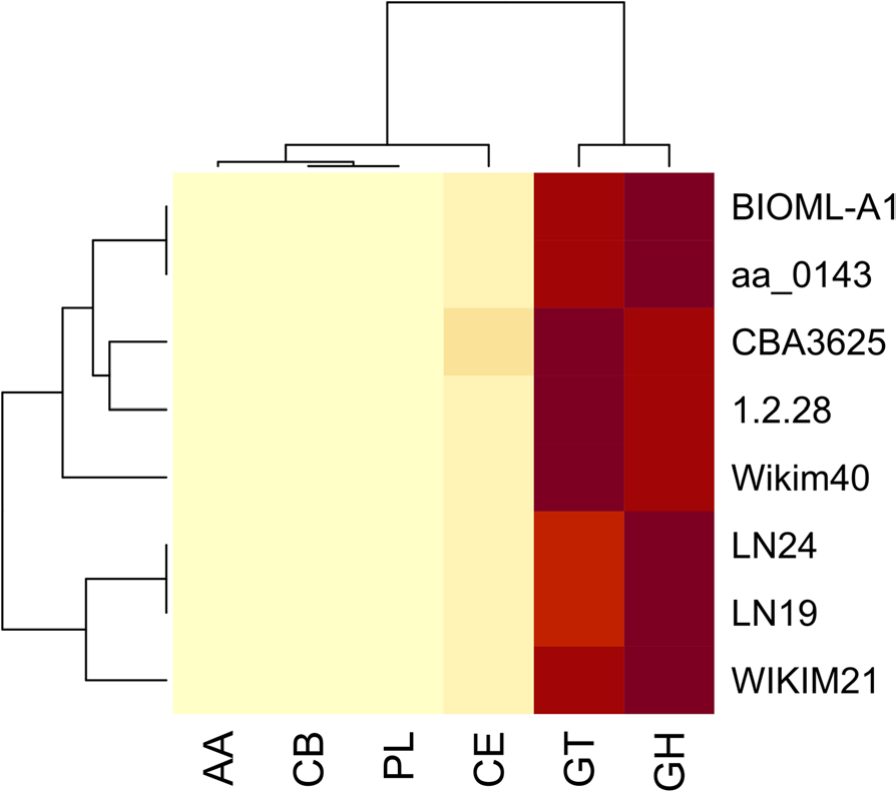
Heatmap of CAZymes distribution and clustering across eight *Ln. lactis* genomes. The color gradient from lighter to darker colours represents the abundance of CAZymes found in each genome. GH: Glycoside hydrolase, GT: Glycosyltransferase, CE: Carbohydrate esterase, AA: Auxiliary activity, CBM: Carbohydrate binding module. R programming language (version 4.1.1) [20] was used to draw the heatmap

Heatmap representation of CAZymes revealed five distinct clades. The amount of GH found was similar within the first and fifth clades (from bottom to up). The concentration of the GT was also found to be similar across the first and fifth clades. However, the highest number of GT family CAZymes does exist in clades three and four. All strains carried a similar amount of CE, except CBA3625, which had the largest number of CE family CAZymes (Fig. 7). The aa_0143 and BIOML-A1 are very similar in the number of enzymes they carry (Fig. 7). Interestingly, WIKIM21 shared the same clade with LN19 and LN24.

**Fig. 8:**
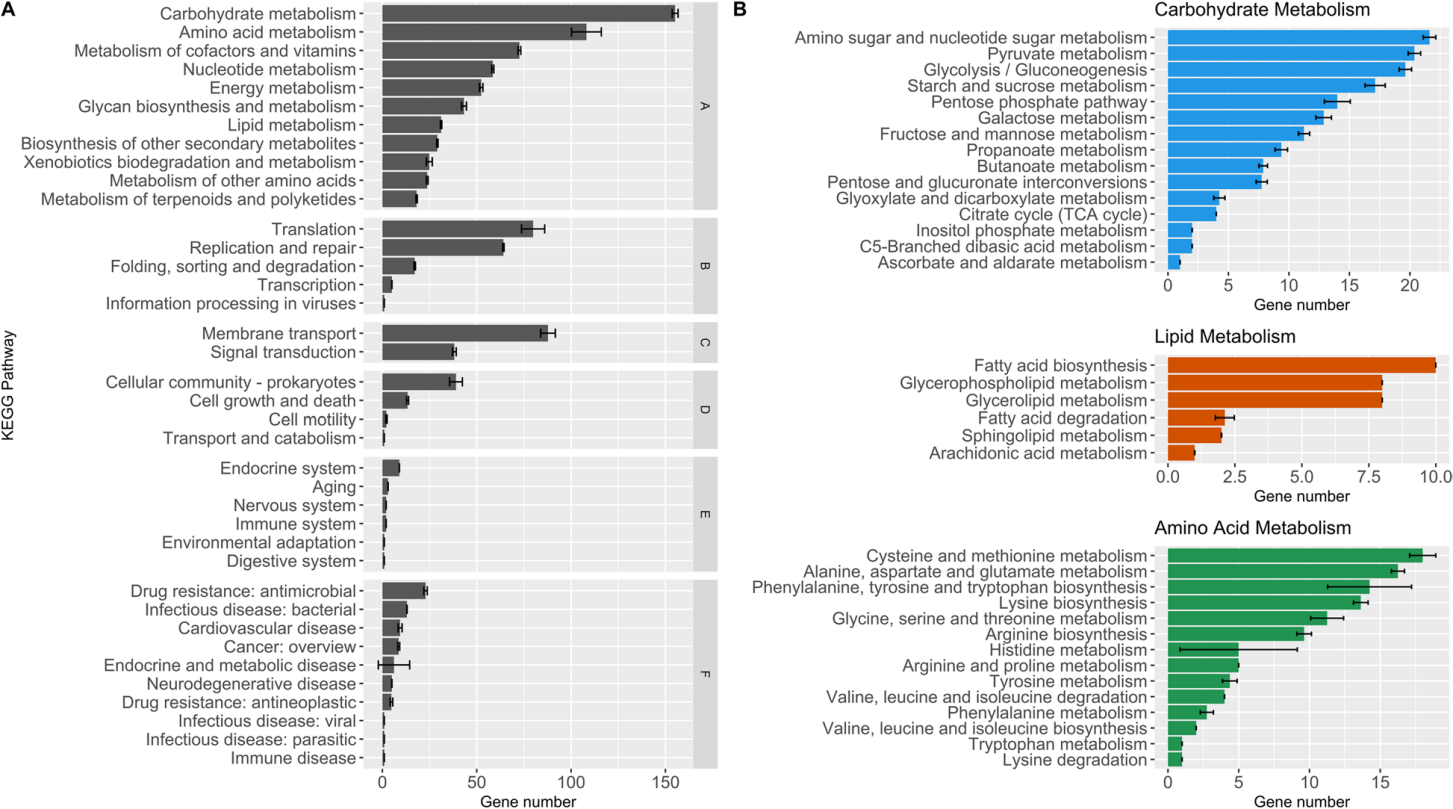
KEGG functional annotations a) detailed representation of functional classes belonging to six main functional categories b) subcategories of carbohydrate, lipid, and amino acid metabolism. Functional categories: **A** Metabolism, **B** Genetic Information Processing, **C** Environmental Information Processing, **D** Cellular Processes, **E** Organismal Systems, **F** Human Diseases. R programming language [20] and ggplot2 [21] package were used to create the images based on KAAS-KEGG number of functional categories

The core and pangenomes were annotated using Prokka and assigned to functional categories in KAAS. As expected, the largest pangenome categories include CDS with functions associated with carbohydrate metabolism, amino acid metabolism, membrane transport, translation, and vitamins and cofactors metabolism. Functional genome groups, including the lowest number of CDS fall into organismal systems. The highest number of genes accumulated in carbohydrate metabolism were amino- and nucleotide sugar, pyruvate, glycolysis, starch and sucrose metabolism. Major functional genes associated with lipid metabolism are pertained to fatty acid biosynthesis, glycerophospholipid, and glycerolipid metabolism. Amino acid metabolism mainly consists of cysteine and methionine, alanine, aspartate and glutamate metabolism, and phenylalanine, tyrosine and tryptophan biosynthesis. The highest standard deviation bars were achieved in histidine metabolism, and phenylalanine, tyrosine and tryptophan biosynthesis. The lowest number of genes in amino acid metabolism relates to tryptophan metabolism and lysine degradation (Fig. 8).

### Mobile genetic elements

**Fig. 9:**
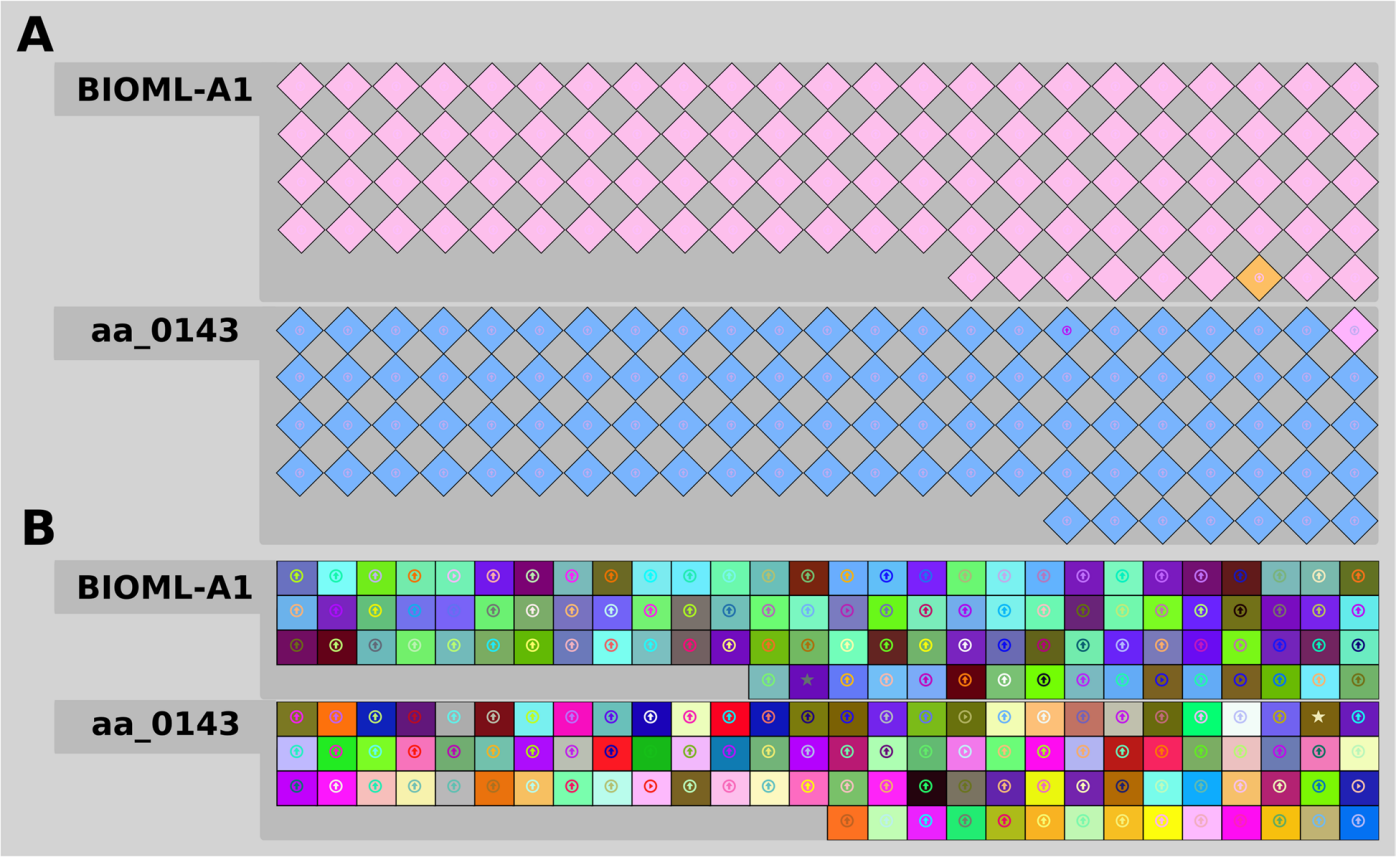
Visual representation and alignment of the repeat (**A**) and spacer (**B**) contents for each identified CRISPR locus. Each diamond corresponds to a CRISPR repeat, while each colored square corresponds to a CRISPR spacer. Unique color combinations show distinct nucleotide composition

Spacers and repeats from entire CRISPR loci were identified using CRISPRviz. Among all strains screened, only aa_0143 and BIOML-A1 harbor a single spacer. The spacer alignment revealed no identical spacer sequences across two strains showing a robust confirmation of evolutionary heterology (Fig. 9B). A similar heterology was also seen in repeat sequence alignments of BIOML-A1 and aa_0143 (Fig. 9A).

Table 2 shows intact and questionable prophage regions in genomes of all *Ln. lactis* strains screened. Prophage analysis results from PHASTER show that six strains (1.2.28, aa_0143, BIOML-A1, CBA3625, LN19, WIKIM21) have intact prophages; three strains (LN19, LN24, and WiKim40) have questionable prophage regions at their genomes. The size of intact and questionable prophages range between 20.8 Kb — 47.4 Kb (34.9 Kb on average) and 9.6 Kb —32.8 Kb (16.5 Kb on average), respectively.

A total of three plasmids were discovered in BIOML-A1, LN19, and LN24, with the former harboring repUS2 and the latter two strain containing rep31 (Table 3). The size of plasmids ranges from 0.66 kb to 1.15 kb. The minimum percent identity of predicted plasmids is 98.8%.

**Table 3:** Putative plasmids and genomic locations across three *Ln. lactis* strains (Strains not shown in the table were not predicted to carry plasmid)

Strain	Plasmid	Identity	Size (kb)	Accession number
BIOML-A1	repUS2	98.8	0.663	BFU30316
LN19	rep31	99.05	1.154	DQ489739
LN24	rep31	99.05	1.154	DQ489739

### Bacteriocins

Analysis of *Ln. lactis* genomes with BAGEL4 showed a single type of bacteriocin “Lactoccoccin 972” exists in all genomes except CBA3625, which indicates potential antimicrobial characteristics of *Ln. lactis* strains. All results taken from BAGEL4 are checked with NCBI protein BLAST to validate bacteriocins.

**Fig. 10:**
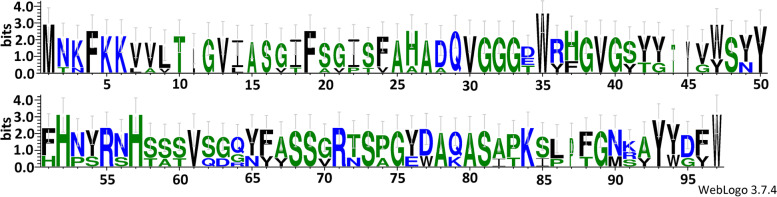
The amino acid sequence logo of Lactococcin 972

Weblogo results show that amino acid sequences of Lactococcin 972 discovered in the *Ln. lactis* genomes in the present study were similar. A strong MNKFKKK motif is identified in N-terminus of Lactococcin 972 (Fig. 10).

## Discussion

In the present study, we genomically evaluated the *Ln. lactis* species and focused on eight strains representing the human gastrointestinal tract and fermented foods microbiomes. GC content is typical for low GC LAB. The proportion of hypothetical genes indicates that there is still more to uncover about *Leuconostoc lactis*. After extracting the genome of *Ln. lactis,* we conducted a global phylogeny of twenty-nine genomes (Fig. 1). This analysis predicted a remarkable diversity between *Ln. lactis* strains. Nine distinct clades were determined.

**Fig. 11:**
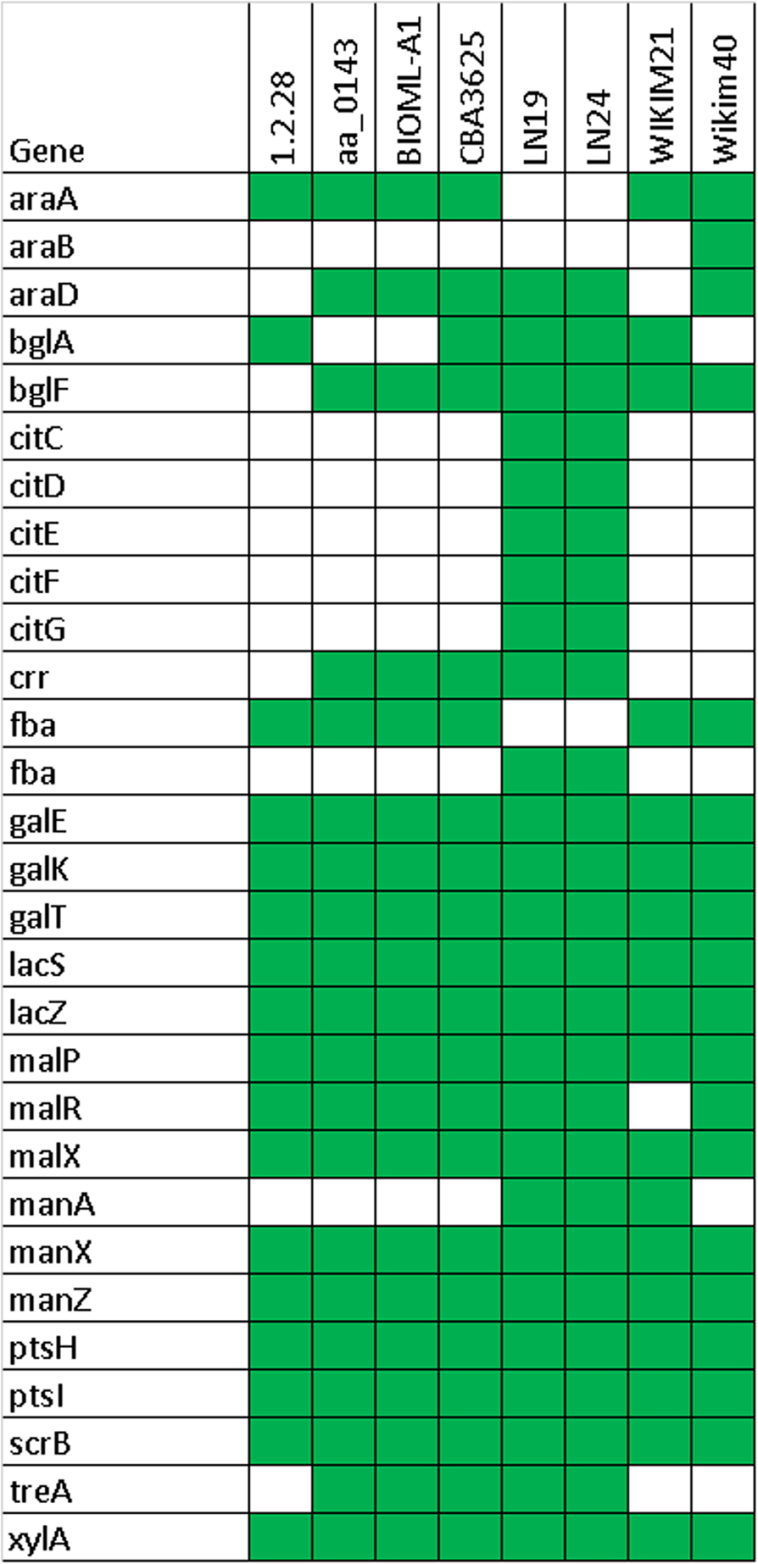
Putative genetic potential of eight *Ln. lactis* strains for carbohydrate metabolism based on predicted transporters or enzymes' presence (green) or absence (white)

Interestingly, WiKim40 was isolated from kimchi, and its clade members were isolated from human feces (Figure S1). This perhaps indicates that *Ln. lactis* enters the gut microbiome through food sources. The genome analysis clearly segregated leuconostocs by species, subspecies, and allowed intra-species and intra-strain differentiations [4]. Generally, dairy isolates of LN19 and LN24, kimchi isolates of CBA3625, WIKIM21, WiKim40, human gastrointestinal isolates of aa_0143 and BIOML-A1, and cucumber fermentation brine isolate of 1.2.28 exist in closely related clades (Fig. 2). Only dairy-associated strains lack the arabinose metabolism genes such as *araA,* while the rest of the strains harbored that gene, which perhaps relates to the fact that no arabinose sugar exists in dairy environments. Apart from aa_0143, BIOML-A1, LN19, and LN24 remaining four strains were isolated from fermented plant materials where arabinose sugar is part of the composition. From an evolutionary perspective, this shows that repetitive subculturing of LN19 and LN24 in dairy caused the gene loss or gene decay of *araA* due to lack of this sugar in the milk microenvironment (Fig. 11). Similar results were also reported for *Lactobacillus (L.) casei* supragenome that the strain isolated from milk vs silage material had a different carbohydrate fermentation profile [23]. For example, the dairy strain could utilize lactose but not inulin, whereas silage isolate is lactose negative but inulin positive. We also found that *bglF* gene encoding glucose/b-glucoside family PTS transporter EIICBA did exist in all strains except cucumber fermentation brine isolate of 1.2.28. However, the *crr* gene encoding glucose/b-glucoside family PTS transporter EIIA did exist in all strains except for WIKIM21, WiKim40, and 1.2.28. The *celC* gene encoding cellobiose-diacetyl chitobiose family PTS transporter EIIA was not found in any strains tested (Fig. 11). In a study comparing 17 *Ln. carnosum* strains isolated from meat reported the presence of *celA*, *celB*, and *celC* genes [17], which implies intra-species diversity within *Leuconostoc* genus.

Obst et al. (1995) [24] reported that *Leuconostoc* strains isolated from dairy are reported to acquire plasmid-encoded *LacLM* through horizontal gene transfer to adapt milk microenvironment. Interestingly, all eight strains tested in the present work were found to be carrying *lacS* and *lacZ* genes regardless of their isolation source. Only dairy-associated strains of LN19 and LN24 harbored *cit* operon composed of *citC, citD, citE, citF*, and *citG* (Fig. 11). The lack of citrate uptake and utilization related genes in non-dairy associated *Ln. lactis* strains show evidence of prolonged degenerative evolution, perhaps due to a long period of proliferation in non-dairy niches [4] where no citrate exists.

Choline transport was predicted to be existing only in LN19 and LN24. These strains perhaps utilize choline, which is available in a wide range of milk products [25], to combat osmotic stress conditions such as high salt-in-moisture content of cheese. However, all strains were predicted to carry betaine transport genes that are functional in osmoprotectant activity [26].

The absence of genes encoding antibiotic resistance perhaps relates to the specificity of *Ln. lactis* for dairy and plant-based fermentation matrices secluded this species under limited selective pressure in such a microenvironment as antibiotic usage in plant, and dairy production is restricted. Safety of *Ln. lactis* is a critical phenomenon given that a considerable number of bacteria belong to this species which are ingested as foods for example cheese and kimchi. Another critical safety factor is biogenic amine production by decarboxylase genes which were also not found in dairy-associated strains where health concern is more pronounced than plant materials [17].

*Ln. lactis* shows evidence of prolonged evolutionary degeneration, perhaps due to long and repetitive periods of proliferation in milk and fermented plant materials such as kimchi and cucumber fermentation. Dairy-associated *Ln. lactis* strains appeared to be evolved alongside *L. helveticus* and *L. sanfranciscensis.* IS3 family showing significant sequence alignment in kimchi, fecal material, dairy material, and cucumber fermentation brine isolates indicating this IS element belonged to *Weissella cibaria* and was likely imported via horizontal gene transfer (Table S1). Dairy isolates perhaps evolved with *L. helveticus* because it is heavily utilized as adjunct and starter culture in the dairy industry for flavor and acid development in cheese [27]. LN19 and LN24 were predicted to carry the IS30 family proposing this IS element was received from *L. helveticus* (Table S1).

Interestingly, both WIKIM21 and WiKim40 contained IS elements predicted to be originating from *L. helveticus* which indicates high adaptability to grow in various micro-niches due to its capability to ferment a broad range of carbohydrates and it was also isolated from plant materials [28].

We did not come across any study describing bacteriocin biosynthesis in *Ln. lactis* strains. In the present study, Lactococcin 972, a homodimeric bacteriocin that targets lactoccal strains, was found in seven *Ln. lactis* strains. It was isolated from *Lactococcus lactis* subsp. *lactis* IPLA 972 in 1996, and unlike other bacteriocins, Lactococcin 972 does not primarily target cell membrane [29]. The bacteriocin synthesis potential of *Ln. lactis* is reported for the first time in the present study. Therefore, the screening for unique antimicrobials needs further studies due to diverse microbial ecosystems occupied by *Ln. lactis* strains and abundance of CDS without any function assigned.

It was reported that Lactococcin 972 shows a narrow and specific antimicrobial spectrum similar to Lactococcin Q, a dipeptide bacteriocin biosynthesized by *Lactococcus lactis* QU 4, which possesses antagonistic activity only against *Lactococcus lactis* strains [30]. This might be a competitive inhibition strategy that *Ln. lactis* developed to perhaps inhibit *Lactococcus lactis* where they usually coexist together, especially in dairy applications where *Lactococcus lactis* starter cultures are heavily utilized [31]. This could be explained by competitive exclusion, where two strains competing for the same nutrient cannot stably coexist; thus, a competitive strain always dominates its competitor and forces evolutionary modification and shifts to another niche or extinction [32].

In all strains screened, CRISPR-Cas system was found in aa_0143 and BIOML-A1, which have human fecal material origin. Cheese dead vats (i.e. slow or no milk acidification) cause huge economical loss for dairy industry due to bacteriophage infection of starter cultures such as *Lactococcus lactis*, *Streptococcus thermophilus*, and *Ln. lactis* [33]. The knowledge on *Ln. lactis*’ CRISPR-Cas could be further explored in fermented dairy foods biotechnology to protect and reduce bacteriophage infection of *Ln. lactis* dairy starter cultures (LN19 and LN24) for preventing economic loss in industry and conferring robust bioprocesses. The CRISPR-Cas in *Ln. lactis* strains described in the present study should further encounter functional assessment for investigating their utilization in microbial engineering against bacteriophage resistance to confer phage immunity to starter cultures.

## Conclusion

This study aimed to boost the available fundamental knowledge on *Leuconostoc lactis*, a microorganism that plays an important role in industrial food fermentations. Global phylogeny on twenty-nine *Ln. lactis* strains revealed a great deal of diversity. A comparative whole-genome sequence analysis was performed on eight strains representing the human gastrointestinal tract and fermented foods. Comparative genome analysis showed all strains possess mobile genomic elements, namely insertion sequences (IS). CRISPR-Cas system was discovered in each of aa_0143 and BIOML-A1. All strains except CBA3625, LN 24, and WiKim40 harbor at least one intact putative prophage region. Apart from CBA3625, all strains encode genes functional for putative Lactococcin 972 biosynthesis. Metabolic differences according to strain isolation source were found between dairy and non-dairy (plant material) associated strains. For instance, plant-associated strains could utilize plant-based sugar arabinose, whereas dairy strains could not. However, dairy-associated strains were able to metabolize citrate though plant isolates could not, which perhaps relates to the loss of citrate uptake and utilization genes part of evolutionary adaptation due to repetitive growth of strains in plant fermentations where no citrate exists. We hope to contribute to setting the pipeline for future research and convey feasible data for better quality industrial biomanufacturing via dilating comparative genomic characterization of *Ln. lactis*.

## Methods

We conducted a global phylogeny of *Ln. lactis* using 29 available genomes in NCBI based on the glycolysis gene “phosphoglucomutase”, which confers a high degree of granularity [34]. No phosphoglucomutase gene was available in four out of thirty-three *Ln. lactis* strains deposited to NCBI. Upon extracting the phosphoglucomutase gene sequence, nucleotide sequences were aligned using MUSCLE [35]. Trees were then constructed using RaxML (GTR, bootstrapping using 100 replicates). Phylogenetic tree of the RaxML result was drawn with Interactive Tree of Life online tool [36]. Next, we run whole-genome based comparisons by using BRIG and Mauve [37] with the following genomes: 1.2.28, aa_0143, BIOML-A1, CBA3625, LN19, LN24, WIKIM21, and WiKim40. These genomes were chosen because of their closed genome status or as selected representatives of distinctive phylogenetic clades from Fig. 1 representing human gastrointestinal tract or fermented foods microbiomes. Whole genomes of eight chosen *Ln. lactis* imported from the database of NCBI Genbank [18]. They are available with the following accession numbers: GCA_018993775.1, GCA_004167235.1, GCA_009678855.1, GCA_007954605.1, GCA_002092595.1, GCA_002092695.1, GCA_001411775.1, GCA_001698145.1. Genomes of those *Ln. lactis* strains were first merged into a single contig using AWK application and annotated with Prokka (version 1.14.5) [38] with the following flags: –kingdom Bacteria. The core- and pan-genomes were analyzed using Roary (version 3.13.0) by feeding Prokka results to Roary [39] with the following flags: –e –n –v –r.

Genomes were clustered, and phylogenetic trees based on whole-genome sequence and 16S rDNA were created on TYGS with default settings (https://tygs.dsmz.de) [40]. The calculation of orthologous average nucleotide identity values (OrthoANI) was performed by OrthoANI tool v0.93.1 [41]. All genomes were aligned and visualized with BLAST Ring Image Generator (BRIG) against CBA3625 as the reference genome [42]. BRIG image was created with the following options: upper percent identity threshold of 90%, lower percent identity threshold of 70%, and ring size of 30. In addition to genome alignments, GC content, GC skew, and prophage regions were mapped on the BRIG image.

Carbohydrate active enzyme (CAZyme) related genes were identified with the CAZy database (v10) in dbCAN server (https://bcb.unl.edu/dbCAN2/index.php) [43] by HMMER version 3.3.32 [44] according to suggested protocol of dbCAN. Results of the CAZYme analysis were classified based on the suggested threshold minimum 0.35 coverage and E-value 1e-15 by Oliviera et al. (2022) [45]. Then, *Ln. lactis* strains were classified based on the number of CAZYmes they harbored in their genomes. Functional annotation of the genomes and distribution of metabolic pathways were performed with KEGG Automatic Annotation Server (KAAS) by selecting prokaryotes as a representative set and bi-directional best hit (BBH) as the assignment method [46]. KEGG Mapper tool was utilized to identify the number of genes associated with functional classes and metabolic pathways [47, 48].

CRISPRviz [49] tool was used to identify, align, and visualize CRISPR loci containing spacers and repeats. In order to identify plasmids and their region in genomes, PlasmidFinder (version 2.0.1) was utilized [50, 51]. The discovery of potential bacteriocins and bacteriocin expressing regions in genomes was performed using BAGEL4 [52]. The potential bacteriocins were analyzed with NCBI Protein BLAST [53] to double-check BAGEL4 results. Then, sequence logo of confirmed bacteriocin was performed with WebLogo tool [54, 55]. Prophage regions located on genomes were identified with Phage Search Tool Enhanced Release (PHASTER) [56]. ISfinder tool [57] was utilized to identify insertion sequences in genomes. Antimicrobial resistance genes were screened by Comprehensive Antibiotic Resistance Database (CARD), a web-based tool [58].

## Supplementary Information

**Additional file 1:** **Fig. S1.** Phylogenetic tree of eight *Leuconostoc lactis* strains based on whole genome sequences. **Fig. S2. **Whole genome comparison of Mauve alignments of eight *Ln. lactis* whole genomes. NCBI accession numbers of the given strains can be found in Table 1. **Table S1.** Putative IS elements of eight *Ln. lactis* strains

## Data Availability

The genomes analyzed in the current study are available in the NCBI GenBank repository under the following accession numbers: GCA_018993775.1 (1.2.28), GCA_015551285.1 (1001262B_160229_C9), GCA_004167235.1 (aa_0143), GCA_009678855.1 (BIOML-A1), GCA_007954625.1 (CBA3622), GCA_007954605.1 (CBA3625), GCA_007954665.1 (CBA3626), GCA_002287365.1 (CCK940), GCA_019656035.1 (JCM 6123), GCA_014651235.1 (JCM 6123), GCA_000709265.1 (KACC 91922), GCA_000185085.2 (KCTC 3528), GCA_000179875.1 (KCTC 3773), GCA_002092595.1 (LN19), GCA_002092695.1 (LN24), GCA_020708945.1 (MSK.22.137), GCA_020708975.1 (MSK.22.141), GCA_006539105.1 (NBRC 12455), GCA_014050705.1 (SBC001), GCA_002386625.1 (UBA4605), GCA_002386555.1 (UBA4610), GCA_002425565.1 (UBA5566), GCA_002425485.1 (UBA5570), GCA_002420925.1 (UBA5657), GCA_002453615.1 (UBA6751), GCA_003521925.1 (UBA8466), GCA_003529125.1 (UBA8811), GCA_001411775.1 (WIKIM21), GCA_001698145.1 (WiKim40).
