# Supplementary material for: Comparative genomics of Leuconostoc lactis strains isolated from human gastrointestinal system and fermented foods microbiomes
Source: BMC Genom Data. 2022 Aug 2;23:61. doi: 10.1186/s12863-022-01074-6 (PMC9344693; doi:10.1186/s12863-022-01074-6)
Supplement: Supplementary file 1 — Additional file 1: Fig. S1. Phylogenetic tree of eight Leuconostoc lactis strains based on whole genome sequences. Fig. S2. Whole genome comparison of Mauve alignments of eight Ln. lactis whole genomes. NCBI accession numbers of the given strains can be found in Table 1. Table S1. Putative IS elements of eight Ln. lactis strains [file 12863_2022_1074_MOESM1_ESM.docx]

**Supplementary Files**

**Comparative genomics of *Leuconostoc lactis* strains isolated from human gastrointestinal system and fermented foods microbiomes**

**Ismail Gumustop^1^, Fatih Ortakci^1^***

**^1^BioEngineering Department, Faculty of Life and Natural Sciences, Abdullah Gul University, Kayseri, TR**

***Corresponding author:** [**fatih.ortakci@agu.edu.tr**](mailto:fatih.ortakci@agu.edu.tr) **(FO)**


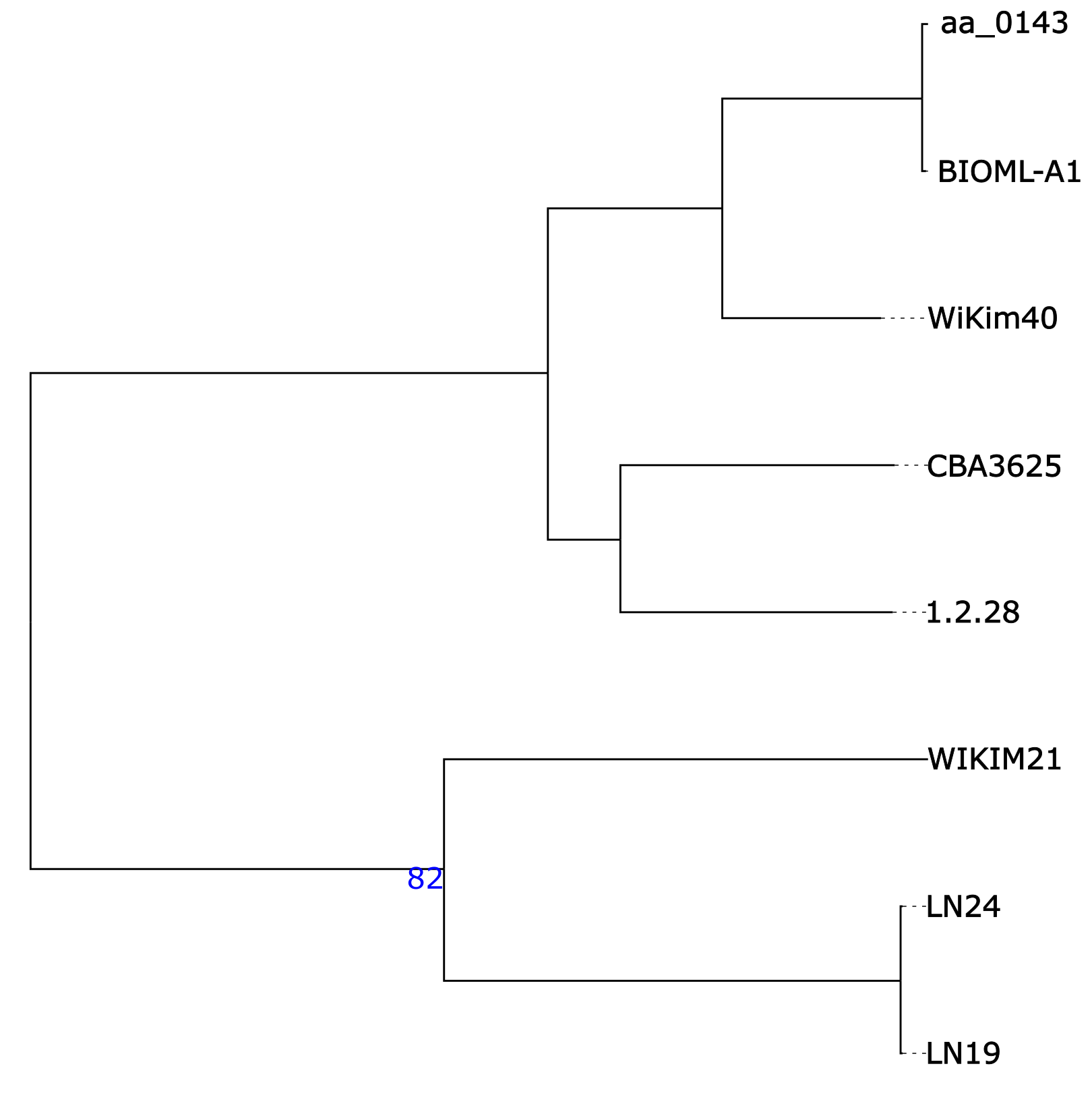


Figure S1. Phylogenetic tree of eight *Leuconostoc lactis* strains based on whole genome sequences

The phylogenetic analysis of eight *Ln. lactis* strains the lower phylogenetic distance shows close relationship between strains. BIOML-A1 and aa_0143 are very similar and they were also isolated from the same origin. Similarly, LN19 and LN24 shares same node and both are dairy associated *Ln. lactis strains*. However, genomes of WiKim40 and BIOML-A1 strains are very similar despite their origin.


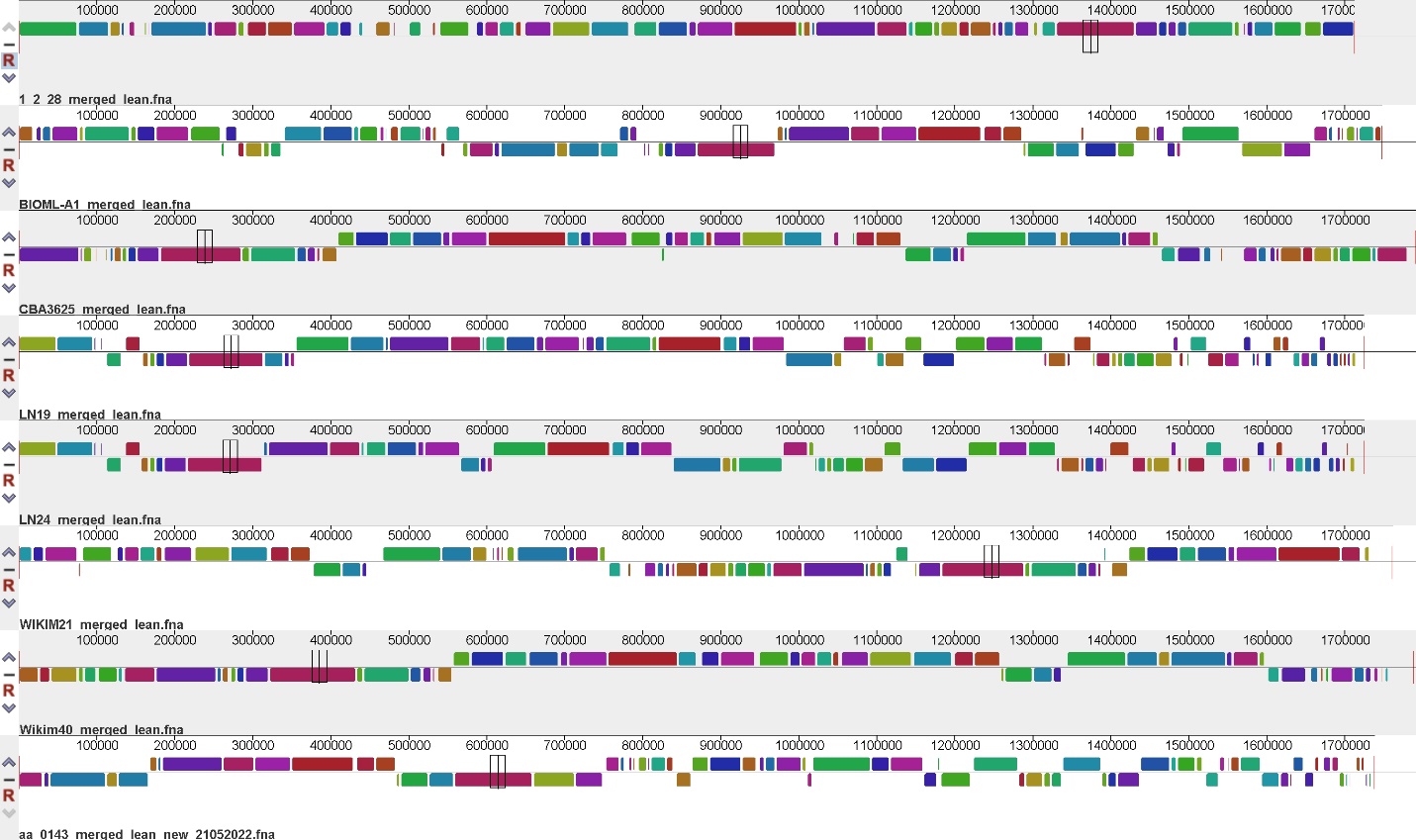


Figure S2. Whole genome comparison of Mauve alignments of all *Ln. lactis* whole genomes. NCBI accession numbers of the given strains can be found in Table 1.

Table S1. Putative IS elements of eight *Ln. lactis* strains.

| Strain | Sequences producing significant alignments | IS Family | Group | Origin | Score (bits) | E. value |
| --- | --- | --- | --- | --- | --- | --- |
| 1.2.28 | [IS1070](https://isfinder.biotoul.fr/scripts/ficheIS.php?name=IS1070) | IS30 |  | [Leuconostoc lactis](http://www.ncbi.nlm.nih.gov/Taxonomy/Browser/wwwtax.cgi?name=Leuconostoc+lactis) | [1933](https://isfinder.biotoul.fr/blast/resultat.php?id=phpNaq9cE&title=1.2.28_IS_Finder_25052022&prog=blastn#BL_ORD_ID:31) | 0 |
| 1.2.28 | [ISWci1](https://isfinder.biotoul.fr/scripts/ficheIS.php?name=ISWci1) | IS3 | IS150 | [Weissella cibaria](http://www.ncbi.nlm.nih.gov/Taxonomy/Browser/wwwtax.cgi?name=Weissella+cibaria) | [480](https://isfinder.biotoul.fr/blast/resultat.php?id=phpNaq9cE&title=1.2.28_IS_Finder_25052022&prog=blastn#BL_ORD_ID:5326) | 4E-132 |
| 1.2.28 | [ISWci2](https://isfinder.biotoul.fr/scripts/ficheIS.php?name=ISWci2) | IS3 | IS3 | [Weissella cibaria](http://www.ncbi.nlm.nih.gov/Taxonomy/Browser/wwwtax.cgi?name=Weissella+cibaria) | [97.6](https://isfinder.biotoul.fr/blast/resultat.php?id=phpNaq9cE&title=1.2.28_IS_Finder_25052022&prog=blastn#BL_ORD_ID:5347) | 6E-17 |
| aa_0143 | [ISWci1](https://isfinder.biotoul.fr/scripts/ficheIS.php?name=ISWci1) | IS3 | IS150 | [Weissella cibaria](http://www.ncbi.nlm.nih.gov/Taxonomy/Browser/wwwtax.cgi?name=Weissella+cibaria) | [609](https://isfinder.biotoul.fr/blast/resultat.php?id=phpEeuEyH&title=aa_0143_25052022&prog=blastn#BL_ORD_ID:5326) | 7E-171 |
| aa_0143 | [ISWco1](https://isfinder.biotoul.fr/scripts/ficheIS.php?name=ISWco1) | IS3 | IS150 | [Weissella confusa](http://www.ncbi.nlm.nih.gov/Taxonomy/Browser/wwwtax.cgi?name=Weissella+confusa) | [464](https://isfinder.biotoul.fr/blast/resultat.php?id=phpEeuEyH&title=aa_0143_25052022&prog=blastn#BL_ORD_ID:5342) | 2E-127 |
| aa_0143 | [ISWci2](https://isfinder.biotoul.fr/scripts/ficheIS.php?name=ISWci2) | IS3 | IS3 | [Weissella cibaria](http://www.ncbi.nlm.nih.gov/Taxonomy/Browser/wwwtax.cgi?name=Weissella+cibaria) | [147](https://isfinder.biotoul.fr/blast/resultat.php?id=phpEeuEyH&title=aa_0143_25052022&prog=blastn#BL_ORD_ID:5347) | 7E-32 |
| BIOML-A1 | [ISWci1](https://isfinder.biotoul.fr/scripts/ficheIS.php?name=ISWci1) | IS3 | IS150 | [Weissella cibaria](http://www.ncbi.nlm.nih.gov/Taxonomy/Browser/wwwtax.cgi?name=Weissella+cibaria) | [617](https://isfinder.biotoul.fr/blast/resultat.php?id=phpFieoQM&title=BIOML-A1_25052022&prog=blastn#BL_ORD_ID:5326) | 3E-173 |
| BIOML-A1 | [ISWco1](https://isfinder.biotoul.fr/scripts/ficheIS.php?name=ISWco1) | IS3 | IS150 | [Weissella confusa](http://www.ncbi.nlm.nih.gov/Taxonomy/Browser/wwwtax.cgi?name=Weissella+confusa) | [464](https://isfinder.biotoul.fr/blast/resultat.php?id=phpFieoQM&title=BIOML-A1_25052022&prog=blastn#BL_ORD_ID:5342) | 2E-127 |
| BIOML-A1 | [ISWci2](https://isfinder.biotoul.fr/scripts/ficheIS.php?name=ISWci2) | IS3 | IS3 | [Weissella cibaria](http://www.ncbi.nlm.nih.gov/Taxonomy/Browser/wwwtax.cgi?name=Weissella+cibaria) | [147](https://isfinder.biotoul.fr/blast/resultat.php?id=phpFieoQM&title=BIOML-A1_25052022&prog=blastn#BL_ORD_ID:5347) | 7E-32 |
| CBA3625 | [IS1070](https://isfinder.biotoul.fr/scripts/ficheIS.php?name=IS1070) | IS30 |  | [Leuconostoc lactis](http://www.ncbi.nlm.nih.gov/Taxonomy/Browser/wwwtax.cgi?name=Leuconostoc+lactis) | [1853](https://isfinder.biotoul.fr/blast/resultat.php?id=php9ZUC0m&title=CBA3625&prog=blastn#BL_ORD_ID:31) | 0 |
| CBA3625 | [ISWci1](https://isfinder.biotoul.fr/scripts/ficheIS.php?name=ISWci1) | IS3 | IS150 | [Weissella cibaria](http://www.ncbi.nlm.nih.gov/Taxonomy/Browser/wwwtax.cgi?name=Weissella+cibaria) | [801](https://isfinder.biotoul.fr/blast/resultat.php?id=php9ZUC0m&title=CBA3625&prog=blastn#BL_ORD_ID:5326) | 0 |
| CBA3625 | [ISLll1](https://isfinder.biotoul.fr/scripts/ficheIS.php?name=ISLll1) | IS982 |  | [Lactococcus lactis](http://www.ncbi.nlm.nih.gov/Taxonomy/Browser/wwwtax.cgi?name=Lactococcus+lactis) | [664](https://isfinder.biotoul.fr/blast/resultat.php?id=php9ZUC0m&title=CBA3625&prog=blastn#BL_ORD_ID:582) | 0 |
| CBA3625 | [IS982B](https://isfinder.biotoul.fr/scripts/ficheIS.php?name=IS982B) | IS982 |  | [Lactococcus lactis](http://www.ncbi.nlm.nih.gov/Taxonomy/Browser/wwwtax.cgi?name=Lactococcus+lactis) | [656](https://isfinder.biotoul.fr/blast/resultat.php?id=php9ZUC0m&title=CBA3625&prog=blastn#BL_ORD_ID:454) | 0 |
| CBA3625 | [IS982C](https://isfinder.biotoul.fr/scripts/ficheIS.php?name=IS982C) | IS982 |  | [Lactococcus lactis](http://www.ncbi.nlm.nih.gov/Taxonomy/Browser/wwwtax.cgi?name=Lactococcus+lactis) | [648](https://isfinder.biotoul.fr/blast/resultat.php?id=php9ZUC0m&title=CBA3625&prog=blastn#BL_ORD_ID:455) | 0 |
| CBA3625 | [IS982](https://isfinder.biotoul.fr/scripts/ficheIS.php?name=IS982) | IS982 |  | [Lactococcus lactis](http://www.ncbi.nlm.nih.gov/Taxonomy/Browser/wwwtax.cgi?name=Lactococcus+lactis) | [640](https://isfinder.biotoul.fr/blast/resultat.php?id=php9ZUC0m&title=CBA3625&prog=blastn#BL_ORD_ID:453) | 2E-180 |
| CBA3625 | [ISLla2](https://isfinder.biotoul.fr/scripts/ficheIS.php?name=ISLla2) | IS982 |  | [Lactococcus lactis](http://www.ncbi.nlm.nih.gov/Taxonomy/Browser/wwwtax.cgi?name=Lactococcus+lactis) | [392](https://isfinder.biotoul.fr/blast/resultat.php?id=php9ZUC0m&title=CBA3625&prog=blastn#BL_ORD_ID:2145) | 8E-106 |
| CBA3625 | [IS-LL6](https://isfinder.biotoul.fr/scripts/ficheIS.php?name=IS-LL6) | IS3 | IS3 | [Lactococcus lactis](http://www.ncbi.nlm.nih.gov/Taxonomy/Browser/wwwtax.cgi?name=Lactococcus+lactis) | [248](https://isfinder.biotoul.fr/blast/resultat.php?id=php9ZUC0m&title=CBA3625&prog=blastn#BL_ORD_ID:581) | 3E-62 |
| CBA3625 | [ISLgar2](https://isfinder.biotoul.fr/scripts/ficheIS.php?name=ISLgar2) | IS982 |  | [Lactococcus garvieae](http://www.ncbi.nlm.nih.gov/Taxonomy/Browser/wwwtax.cgi?name=Lactococcus+garvieae) | [246](https://isfinder.biotoul.fr/blast/resultat.php?id=php9ZUC0m&title=CBA3625&prog=blastn#BL_ORD_ID:4093) | 1E-61 |
| CBA3625 | [ISWci2](https://isfinder.biotoul.fr/scripts/ficheIS.php?name=ISWci2) | IS3 | IS3 | [Weissella cibaria](http://www.ncbi.nlm.nih.gov/Taxonomy/Browser/wwwtax.cgi?name=Weissella+cibaria) | [139](https://isfinder.biotoul.fr/blast/resultat.php?id=php9ZUC0m&title=CBA3625&prog=blastn#BL_ORD_ID:5347) | 2E-29 |
| LN19 | [ISLhe30](https://isfinder.biotoul.fr/scripts/ficheIS.php?name=ISLhe30) | IS30 |  | [Lactobacillus helveticus](http://www.ncbi.nlm.nih.gov/Taxonomy/Browser/wwwtax.cgi?name=Lactobacillus+helveticus) | [1622](https://isfinder.biotoul.fr/blast/resultat.php?id=phpQ9fpyl&title=LN19_25052022&prog=blastn#BL_ORD_ID:5403) | 0 |
| LN19 | [IS1070](https://isfinder.biotoul.fr/scripts/ficheIS.php?name=IS1070) | IS30 |  | [Leuconostoc lactis](http://www.ncbi.nlm.nih.gov/Taxonomy/Browser/wwwtax.cgi?name=Leuconostoc+lactis) | [1263](https://isfinder.biotoul.fr/blast/resultat.php?id=phpQ9fpyl&title=LN19_25052022&prog=blastn#BL_ORD_ID:31) | 0 |
| LN19 | [IS153](https://isfinder.biotoul.fr/scripts/ficheIS.php?name=IS153) | IS3 | IS3 | [Lactobacillus sanfranciscensis](http://www.ncbi.nlm.nih.gov/Taxonomy/Browser/wwwtax.cgi?name=Lactobacillus+sanfranciscensis) | [1007](https://isfinder.biotoul.fr/blast/resultat.php?id=phpQ9fpyl&title=LN19_25052022&prog=blastn#BL_ORD_ID:218) | 0 |
| LN19 | [ISWci2](https://isfinder.biotoul.fr/scripts/ficheIS.php?name=ISWci2) | IS3 | IS3 | [Weissella cibaria](http://www.ncbi.nlm.nih.gov/Taxonomy/Browser/wwwtax.cgi?name=Weissella+cibaria) | [672](https://isfinder.biotoul.fr/blast/resultat.php?id=phpQ9fpyl&title=LN19_25052022&prog=blastn#BL_ORD_ID:5347) | 0 |
| LN19 | [ISWci1](https://isfinder.biotoul.fr/scripts/ficheIS.php?name=ISWci1) | IS3 | IS150 | [Weissella cibaria](http://www.ncbi.nlm.nih.gov/Taxonomy/Browser/wwwtax.cgi?name=Weissella+cibaria) | [505](https://isfinder.biotoul.fr/blast/resultat.php?id=phpQ9fpyl&title=LN19_25052022&prog=blastn#BL_ORD_ID:5326) | 7E-140 |
| LN19 | [IS1062](https://isfinder.biotoul.fr/scripts/ficheIS.php?name=IS1062) | IS30 |  | [Enterococcus faecalis](http://www.ncbi.nlm.nih.gov/Taxonomy/Browser/wwwtax.cgi?name=Enterococcus+faecalis) | [476](https://isfinder.biotoul.fr/blast/resultat.php?id=phpQ9fpyl&title=LN19_25052022&prog=blastn#BL_ORD_ID:1547) | 6E-131 |
| LN19 | [IS1165](https://isfinder.biotoul.fr/scripts/ficheIS.php?name=IS1165) | ISL3 |  | [Leuconostoc mesenteroides](http://www.ncbi.nlm.nih.gov/Taxonomy/Browser/wwwtax.cgi?name=Leuconostoc+mesenteroides) | [406](https://isfinder.biotoul.fr/blast/resultat.php?id=phpQ9fpyl&title=LN19_25052022&prog=blastn#BL_ORD_ID:70) | 5E-110 |
| LN24 | [IS1165](https://isfinder.biotoul.fr/scripts/ficheIS.php?name=IS1165) | ISL3 |  | [Leuconostoc mesenteroides](http://www.ncbi.nlm.nih.gov/Taxonomy/Browser/wwwtax.cgi?name=Leuconostoc+mesenteroides) | [2280](https://isfinder.biotoul.fr/blast/resultat.php?id=phpomX9Xn&title=LN24_25052022&prog=blastn#BL_ORD_ID:70) | 0 |
| LN24 | [ISLhe30](https://isfinder.biotoul.fr/scripts/ficheIS.php?name=ISLhe30) | IS30 |  | [Lactobacillus helveticus](http://www.ncbi.nlm.nih.gov/Taxonomy/Browser/wwwtax.cgi?name=Lactobacillus+helveticus) | [1622](https://isfinder.biotoul.fr/blast/resultat.php?id=phpomX9Xn&title=LN24_25052022&prog=blastn#BL_ORD_ID:5403) | 0 |
| LN24 | [IS1070](https://isfinder.biotoul.fr/scripts/ficheIS.php?name=IS1070) | IS30 |  | [Leuconostoc lactis](http://www.ncbi.nlm.nih.gov/Taxonomy/Browser/wwwtax.cgi?name=Leuconostoc+lactis) | [1209](https://isfinder.biotoul.fr/blast/resultat.php?id=phpomX9Xn&title=LN24_25052022&prog=blastn#BL_ORD_ID:31) | 0 |
| LN24 | [IS153](https://isfinder.biotoul.fr/scripts/ficheIS.php?name=IS153) | IS3 | IS3 | [Lactobacillus sanfranciscensis](http://www.ncbi.nlm.nih.gov/Taxonomy/Browser/wwwtax.cgi?name=Lactobacillus+sanfranciscensis) | [1007](https://isfinder.biotoul.fr/blast/resultat.php?id=phpomX9Xn&title=LN24_25052022&prog=blastn#BL_ORD_ID:218) | 0 |
| LN24 | [ISWci2](https://isfinder.biotoul.fr/scripts/ficheIS.php?name=ISWci2) | IS3 | IS3 | [Weissella cibaria](http://www.ncbi.nlm.nih.gov/Taxonomy/Browser/wwwtax.cgi?name=Weissella+cibaria) | [672](https://isfinder.biotoul.fr/blast/resultat.php?id=phpomX9Xn&title=LN24_25052022&prog=blastn#BL_ORD_ID:5347) | 0 |
| LN24 | [ISWci1](https://isfinder.biotoul.fr/scripts/ficheIS.php?name=ISWci1) | IS3 | IS150 | [Weissella cibaria](http://www.ncbi.nlm.nih.gov/Taxonomy/Browser/wwwtax.cgi?name=Weissella+cibaria) | [505](https://isfinder.biotoul.fr/blast/resultat.php?id=phpomX9Xn&title=LN24_25052022&prog=blastn#BL_ORD_ID:5326) | 7E-140 |
| LN24 | [IS1062](https://isfinder.biotoul.fr/scripts/ficheIS.php?name=IS1062) | IS30 |  | [Enterococcus faecalis](http://www.ncbi.nlm.nih.gov/Taxonomy/Browser/wwwtax.cgi?name=Enterococcus+faecalis) | [476](https://isfinder.biotoul.fr/blast/resultat.php?id=phpomX9Xn&title=LN24_25052022&prog=blastn#BL_ORD_ID:1547) | 6E-131 |
| WIKIM21 | [IS1070](https://isfinder.biotoul.fr/scripts/ficheIS.php?name=IS1070) | IS30 |  | [Leuconostoc lactis](http://www.ncbi.nlm.nih.gov/Taxonomy/Browser/wwwtax.cgi?name=Leuconostoc+lactis) | [1053](https://isfinder.biotoul.fr/blast/resultat.php?id=phpK6eRY1&title=WIKIM21_25052022&prog=blastn#BL_ORD_ID:31) | 0 |
| WIKIM21 | [IS982B](https://isfinder.biotoul.fr/scripts/ficheIS.php?name=IS982B) | IS982 |  | [Lactococcus lactis](http://www.ncbi.nlm.nih.gov/Taxonomy/Browser/wwwtax.cgi?name=Lactococcus+lactis) | [884](https://isfinder.biotoul.fr/blast/resultat.php?id=phpK6eRY1&title=WIKIM21_25052022&prog=blastn#BL_ORD_ID:454) | 0 |
| WIKIM21 | [IS982C](https://isfinder.biotoul.fr/scripts/ficheIS.php?name=IS982C) | IS982 |  | [Lactococcus lactis](http://www.ncbi.nlm.nih.gov/Taxonomy/Browser/wwwtax.cgi?name=Lactococcus+lactis) | [868](https://isfinder.biotoul.fr/blast/resultat.php?id=phpK6eRY1&title=WIKIM21_25052022&prog=blastn#BL_ORD_ID:455) | 0 |
| WIKIM21 | [ISLll1](https://isfinder.biotoul.fr/scripts/ficheIS.php?name=ISLll1) | IS982 |  | [Lactococcus lactis](http://www.ncbi.nlm.nih.gov/Taxonomy/Browser/wwwtax.cgi?name=Lactococcus+lactis) | [852](https://isfinder.biotoul.fr/blast/resultat.php?id=phpK6eRY1&title=WIKIM21_25052022&prog=blastn#BL_ORD_ID:582) | 0 |
| WIKIM21 | [IS982](https://isfinder.biotoul.fr/scripts/ficheIS.php?name=IS982) | IS982 |  | [Lactococcus lactis](http://www.ncbi.nlm.nih.gov/Taxonomy/Browser/wwwtax.cgi?name=Lactococcus+lactis) | [837](https://isfinder.biotoul.fr/blast/resultat.php?id=phpK6eRY1&title=WIKIM21_25052022&prog=blastn#BL_ORD_ID:453) | 0 |
| WIKIM21 | [ISWci1](https://isfinder.biotoul.fr/scripts/ficheIS.php?name=ISWci1) | IS3 | IS150 | [Weissella cibaria](http://www.ncbi.nlm.nih.gov/Taxonomy/Browser/wwwtax.cgi?name=Weissella+cibaria) | [714](https://isfinder.biotoul.fr/blast/resultat.php?id=phpK6eRY1&title=WIKIM21_25052022&prog=blastn#BL_ORD_ID:5326) | 0 |
| WIKIM21 | [ISLla2](https://isfinder.biotoul.fr/scripts/ficheIS.php?name=ISLla2) | IS982 |  | [Lactococcus lactis](http://www.ncbi.nlm.nih.gov/Taxonomy/Browser/wwwtax.cgi?name=Lactococcus+lactis) | [690](https://isfinder.biotoul.fr/blast/resultat.php?id=phpK6eRY1&title=WIKIM21_25052022&prog=blastn#BL_ORD_ID:2145) | 0 |
| WIKIM21 | [ISWci2](https://isfinder.biotoul.fr/scripts/ficheIS.php?name=ISWci2) | IS3 | IS3 | [Weissella cibaria](http://www.ncbi.nlm.nih.gov/Taxonomy/Browser/wwwtax.cgi?name=Weissella+cibaria) | [672](https://isfinder.biotoul.fr/blast/resultat.php?id=phpK6eRY1&title=WIKIM21_25052022&prog=blastn#BL_ORD_ID:5347) | 0 |
| WIKIM21 | [ISLgar2](https://isfinder.biotoul.fr/scripts/ficheIS.php?name=ISLgar2) | IS982 |  | [Lactococcus garvieae](http://www.ncbi.nlm.nih.gov/Taxonomy/Browser/wwwtax.cgi?name=Lactococcus+garvieae) | [434](https://isfinder.biotoul.fr/blast/resultat.php?id=phpK6eRY1&title=WIKIM21_25052022&prog=blastn#BL_ORD_ID:4093) | 2E-118 |
| WIKIM21 | [ISWco1](https://isfinder.biotoul.fr/scripts/ficheIS.php?name=ISWco1) | IS3 | IS150 | [Weissella confusa](http://www.ncbi.nlm.nih.gov/Taxonomy/Browser/wwwtax.cgi?name=Weissella+confusa) | [234](https://isfinder.biotoul.fr/blast/resultat.php?id=phpK6eRY1&title=WIKIM21_25052022&prog=blastn#BL_ORD_ID:5342) | 4E-58 |
| WIKIM21 | [ISLhe30](https://isfinder.biotoul.fr/scripts/ficheIS.php?name=ISLhe30) | IS30 |  | [Lactobacillus helveticus](http://www.ncbi.nlm.nih.gov/Taxonomy/Browser/wwwtax.cgi?name=Lactobacillus+helveticus) | [123](https://isfinder.biotoul.fr/blast/resultat.php?id=phpK6eRY1&title=WIKIM21_25052022&prog=blastn#BL_ORD_ID:5403) | 1E-24 |
| WiKim40 | [ISLhe30](https://isfinder.biotoul.fr/scripts/ficheIS.php?name=ISLhe30) | IS30 |  | [Lactobacillus helveticus](http://www.ncbi.nlm.nih.gov/Taxonomy/Browser/wwwtax.cgi?name=Lactobacillus+helveticus) | [878](https://isfinder.biotoul.fr/blast/resultat.php?id=phpCk4wBT&title=WiKim40&prog=blastn#BL_ORD_ID:5403) | 0 |
| WiKim40 | [ISWci1](https://isfinder.biotoul.fr/scripts/ficheIS.php?name=ISWci1) | IS3 | IS150 | [Weissella cibaria](http://www.ncbi.nlm.nih.gov/Taxonomy/Browser/wwwtax.cgi?name=Weissella+cibaria) | [791](https://isfinder.biotoul.fr/blast/resultat.php?id=phpCk4wBT&title=WiKim40&prog=blastn#BL_ORD_ID:5326) | 0 |
| WiKim40 | [ISLll1](https://isfinder.biotoul.fr/scripts/ficheIS.php?name=ISLll1) | IS982 |  | [Lactococcus lactis](http://www.ncbi.nlm.nih.gov/Taxonomy/Browser/wwwtax.cgi?name=Lactococcus+lactis) | [662](https://isfinder.biotoul.fr/blast/resultat.php?id=phpCk4wBT&title=WiKim40&prog=blastn#BL_ORD_ID:582) | 0 |
| WiKim40 | [IS982B](https://isfinder.biotoul.fr/scripts/ficheIS.php?name=IS982B) | IS982 |  | [Lactococcus lactis](http://www.ncbi.nlm.nih.gov/Taxonomy/Browser/wwwtax.cgi?name=Lactococcus+lactis) | [654](https://isfinder.biotoul.fr/blast/resultat.php?id=phpCk4wBT&title=WiKim40&prog=blastn#BL_ORD_ID:454) | 0 |
| WiKim40 | [IS982C](https://isfinder.biotoul.fr/scripts/ficheIS.php?name=IS982C) | IS982 |  | [Lactococcus lactis](http://www.ncbi.nlm.nih.gov/Taxonomy/Browser/wwwtax.cgi?name=Lactococcus+lactis) | [646](https://isfinder.biotoul.fr/blast/resultat.php?id=phpCk4wBT&title=WiKim40&prog=blastn#BL_ORD_ID:455) | 0 |
| WiKim40 | [IS982](https://isfinder.biotoul.fr/scripts/ficheIS.php?name=IS982) | IS982 |  | [Lactococcus lactis](http://www.ncbi.nlm.nih.gov/Taxonomy/Browser/wwwtax.cgi?name=Lactococcus+lactis) | [638](https://isfinder.biotoul.fr/blast/resultat.php?id=phpCk4wBT&title=WiKim40&prog=blastn#BL_ORD_ID:453) | 8E-180 |
| WiKim40 | [ISWco1](https://isfinder.biotoul.fr/scripts/ficheIS.php?name=ISWco1) | IS3 | IS150 | [Weissella confusa](http://www.ncbi.nlm.nih.gov/Taxonomy/Browser/wwwtax.cgi?name=Weissella+confusa) | [464](https://isfinder.biotoul.fr/blast/resultat.php?id=phpCk4wBT&title=WiKim40&prog=blastn#BL_ORD_ID:5342) | 2E-127 |
| WiKim40 | [ISLla2](https://isfinder.biotoul.fr/scripts/ficheIS.php?name=ISLla2) | IS982 |  | [Lactococcus lactis](http://www.ncbi.nlm.nih.gov/Taxonomy/Browser/wwwtax.cgi?name=Lactococcus+lactis) | [392](https://isfinder.biotoul.fr/blast/resultat.php?id=phpCk4wBT&title=WiKim40&prog=blastn#BL_ORD_ID:2145) | 8E-106 |
| WiKim40 | [IS1165](https://isfinder.biotoul.fr/scripts/ficheIS.php?name=IS1165) | ISL3 |  | [Leuconostoc mesenteroides](http://www.ncbi.nlm.nih.gov/Taxonomy/Browser/wwwtax.cgi?name=Leuconostoc+mesenteroides) | [385](https://isfinder.biotoul.fr/blast/resultat.php?id=phpCk4wBT&title=WiKim40&prog=blastn#BL_ORD_ID:70) | 2E-103 |
| WiKim40 | [IS1070](https://isfinder.biotoul.fr/scripts/ficheIS.php?name=IS1070) | IS30 |  | [Leuconostoc lactis](http://www.ncbi.nlm.nih.gov/Taxonomy/Browser/wwwtax.cgi?name=Leuconostoc+lactis) | [333](https://isfinder.biotoul.fr/blast/resultat.php?id=phpCk4wBT&title=WiKim40&prog=blastn#BL_ORD_ID:31) | 6E-88 |
| WiKim40 | [ISWci2](https://isfinder.biotoul.fr/scripts/ficheIS.php?name=ISWci2) | IS3 | IS3 | [Weissella cibaria](http://www.ncbi.nlm.nih.gov/Taxonomy/Browser/wwwtax.cgi?name=Weissella+cibaria) | [285](https://isfinder.biotoul.fr/blast/resultat.php?id=phpCk4wBT&title=WiKim40&prog=blastn#BL_ORD_ID:5347) | 1E-73 |
| WiKim40 | [ISLgar2](https://isfinder.biotoul.fr/scripts/ficheIS.php?name=ISLgar2) | IS982 |  | [Lactococcus garvieae](http://www.ncbi.nlm.nih.gov/Taxonomy/Browser/wwwtax.cgi?name=Lactococcus+garvieae) | [244](https://isfinder.biotoul.fr/blast/resultat.php?id=phpCk4wBT&title=WiKim40&prog=blastn#BL_ORD_ID:4093) | 4E-61 |
